# The health benefit of physical exercise on COVID-19 pandemic: Evidence from mainland China

**DOI:** 10.1371/journal.pone.0275425

**Published:** 2022-10-12

**Authors:** Ruofei Lin, Xiaoli Hu, Lige Guo, Junpei Huang

**Affiliations:** 1 School of Economics and Management, Tongji University, Shanghai, China; 2 International College of Football, Tongji University, Shanghai, China; Universiti Malaya, MALAYSIA

## Abstract

**Objectives:**

Our study aims to investigate the health benefit of regular physical exercise participation on a series of COVID-19 outcomes including COVID-19 morbidity, mortality, and cure rate.

**Methods:**

Prefecture-level panel data related to physical exercise and the COVID-19 pandemic in China were collected from January 1 to March 17, 2020, (N = 21379). Multiple linear regression was conducted, and the ordinary least squares technique was used to estimate the coefficient.

**Results:**

It was shown that regular sports participation significantly negatively affected COVID-19 morbidity (estimate = -1.1061, p<0.01) and mortality (estimate = -0.3836, p<0.01), and positively affected cure rate (estimate = 0.0448, p<0.01), implying that engaging in physical exercise regularly does have a significant positive effect on COVID-19 outcomes. Then, we explored the heterogeneity of the effect of physical exercise on areas with different risk levels and it was revealed that the effect of physical exercise was more pronounced in high-risk areas in terms of morbidity (estimate = -1.8776, p<0.01 in high-risk areas; estimate = -0.0037, p<0.01 in low-risk areas), mortality (estimate = -0.3982, p<0.01 in high-risk areas; estimate = -0.3492, p<0.01 in low-risk areas), and cure rate (estimate = 0.0807, p<0.01 in high-risk areas; 0.0193 = -0.0037, p<0.05 in low-risk areas).

**Conclusions:**

Our results suggest that regularly engaging in physical exercise before the pandemic has positive health effects, especially in the case of a more severe epidemic. Therefore, we urge readers to actively engage in physical exercise so that we can reduce the risks in the event of a pandemic.

## 1 Introduction

As an ongoing pandemic, the COVID-19 pandemic has so far infected 500 million people and killed more than 6 million people worldwide. On January 30, 2020, it was declared global health emergency by the World Health Organization (WHO), and then a global pandemic on March 11, 2020 [1]. The COVID-19 pandemic has posed huge challenges to economic and social development and people’s daily life around the world. A large number of medical resources are currently occupied, and the lack of such resources has led to excess mortality [2, 3]. In addition, public health measures such as quarantine and isolation, work at home, and school closure, have seriously affected normal social order and economic production, brought huge economic losses [4], and also have caused a negative impact on the quality of life and physical and mental health of populations worldwide [5]. Therefore, intervention measures should be taken to curb the outbreak as much as possible and minimize the adverse consequences of the epidemic.

Despite a range of outbreak mitigation strategies aimed at social distancing in most countries, practices that have been effective in preventing most citizens from becoming infected during the pandemic, such strategies have paradoxically left people with no immunity to the virus and therefore vulnerable to additional waves of infection [6]. In addition, these public health policies have led to the closure of public places such as parks and gyms, resulting in an increase in sedentary behavior and a decrease in physical exercise (PEx) among individuals, which has also adversely impacted people’s immunity. It is believed that the world will not be able to return to pre-pandemic normalcy until safe and effective vaccines are available and global vaccination programs are successfully implemented [7]. Therefore, countries around the world have been actively developing and promoting COVID-19 vaccines since the outbreak of the COVID-19 pandemic. As of April 26, 2022, a cumulative total of 11.55 billion doses of the COVID-19 vaccine has been reported worldwide, with a vaccination rate of 65.16% [8]. In China, as of April 29, 2022, there have been a cumulative total of 334,308,000 doses administered, with a vaccination rate of 88.64%.

However, despite the proven effectiveness of the vaccine in preventing infection and avoiding critical conditions [9, 10], it has become evident that the SARS-CoV-2 virus can mutate very rapidly [11, 12] with strong antibody evasion [13–16]. Besides, there are still potential negative effects of vaccination [17], especially the prevalence of relatively low vaccination rates but high mortality rates in the elderly [18]. Therefore, at this stage, in addition to timely and effective pharmacological interventions, non-pharmacological interventions aimed at improving autoimmunity are also crucial. As an important part of a healthy lifestyle, it is of great theoretical and practical significance to explore the health benefits of physical exercise in the COVID-19 pandemic.

Physical exercise (PEx) is regarded as an important way to promote health, as well as prevent and protect the body from a variety of diseases [19, 20], and it has been shown to have significant health benefits [21]. Likewise, it is an effective treatment for most chronic diseases and has direct, positive effects on both physical and mental health [22]. It is well documented that exercising regularly significantly improves the nervous system [23], bones and muscles [24, 25], cardiovascular [26] and cardiopulmonary functions [27], and cognitive ability [28], and it has inhibitory effects on adverse health outcomes, including premature death [29, 30], cardiovascular disease [30, 31], hypertension [32], stroke [33], osteoporosis [34], type 2 diabetes [35], obesity [36–38], cancer [39], depression [40–42], anxiety [43] and other health outcomes. More importantly, physical exercise can strengthen the immune system [21, 44–46], improve immune function, and reduce the risk, duration, or severity of viral infections [47]. Therefore, in theory, regular physical exercise can play an important role in strengthening the body’s immune system against COVID-19 [48], and individuals can maintain the requisite immunity to fight the novel infection COVID-19 through adhering to a healthy lifestyle [21, 49].

This study aims to investigate the health benefit of physical exercise on COVID-19 morbidity, mortality, and cure rate. It contributes to the extensive literature on assessing the health benefits of physical exercise. A large body of literature has concluded that PEx has a positive influence on physical and mental health, with studies showing that moderate and regular physical exercise is important for preventing premature death [29, 30], reducing obesity [36–38], avoiding chronic disease, such as cardiovascular and cerebrovascular diseases [32], as well as strengthening bones, muscles [25], and the immune system [44–46]. In addition, it can clear one’s mind and make one energetic [50], help relieve mental stress and reduce the incidence of psychological disorders, such as depression and anxiety [40–43, 51]. In contrast to these articles, this paper focuses on the influence of physical exercise on the public health during the COVID-19 pandemic, and we selected COVID-19 morbidity, mortality, and cure rates as health outcomes of interest.

In addition, our research contributes to ongoing emerging literature related to factors that influence the COVID-19 pandemic. Since the COVID-19 outbreak, the factors influencing the COVID-19 pandemic have been well-discussed in a large number of articles, mainly focusing on pharmacological interventions [52, 53], public health interventions [54–57], environmental variables [58–60], demographic characteristics [61, 62], and healthcare resources [63]. In addition, the benefit of physical exercise (or physical activity) on physical and mental health during the pandemic has also been documented [22, 64–71]. In contrast to those investigations, this paper focuses on the effect of engaging in physical exercise before the outbreak on a series of COVID-19 outcomes during the pandemic.

## 2 Method

### 2.1 Study area

In this research, the study area was 279 prefecture-level Chinese cities. All of the provinces in mainland China were included in our analysis, which also included the great majority of prefectural cities. Cities in ethnic minority autonomous zones including Tibet, Xinjiang, Inner Mongolia, and Southwest China were removed because of the significant amount of missing data, which was unreliable and unrepresentative. It is documented that more than 98% of China’s total population and more than 99% of its GDP were represented by the 279 metropolitan cities [72].

### 2.2 Data and variables

In this article, in order to empirically investigate the influence of physical exercise on COVID-19 morbidity, mortality and cure rates, prefectural data were collected daily from January 1, 2020 to March 17, 2020 in mainland China, N = 21379. Regarding epidemic-related data, the daily data of confirmed cases, death, and cure rates used to calculate morbidity, mortality, and cure rates were collected from official releases on the official websites of the national and provincial Health Committee. Regarding PEx-related data, the proportion of regular physical exercise participants was gathered by collecting data from provincially issued National Fitness Report, the National Fitness Development Survey Bulletin, and the National Fitness Action Program. GDP per capita, population density, and total number of publicly operated buses and taxis were obtained from the China Urban Statistical Yearbook. Applying big data mining technology, population migration data utilized to calculate effective distance were gathered from website of Baidu Migration (http://qianxi.baidu.com/), and the search popularity of the term used to characterize residents’ awareness of prevention was obtained from the website of Baidu Index (http://qianxi.baidu.com/). The original data for public health interventions were compiled from provincial and municipal emergency response headquarters for the prevention and control of COVID-19.

#### 2.2.1 Dependent variable: COVID-19 outcomes

The dependent variables in this paper included daily COVID-19 morbidity, mortality rate, and cure rate, calculated as:

$$morbidity_{i,t}=confirm_{i,t}/population_{i}$$ (1)

where *morbidity*_*i*,*t*_ denotes the COVID-19 morbidity rate of city i at date t, *confirm*_*i*,*t*_ signifies the cumulative number of confirmed cases of city i in date t, and *population*_*i*_ gives us the total population of city i.

$$mortality_{i,t}=death_{i,t}/confirm_{i,t}$$ (2)

where *morbidity*_*i*,*t*_ denotes the COVID-19 mortality rate of city i in date t, and *death*_*i*,*t*_ stands for the number of deaths of city i in date t. If the denominator *confirm*_*i*,*t*_ is 0, then *morbidity*_*i*,*t*_ also takes a value of 0.

$$cure\;rate_{i,t}=cure_{i,t}/confirm_{i,t}$$ (3)

where *cure rate*_*i*,*t*_ represents the COVID-19 cure rate of city i in date t, *cure*_*i*,*t*_ means the number of cures of city i in date t. If the denominator *confirm*_*i*,*t*_ is 0, then *cure*_*i*,*t*_ also takes a value of 0.

#### 2.2.2 Independent variable: Physical exercise participation

The independent variable of this paper was the regular physical exercise participation of city i in 2019, that is, the number of people who regularly engage in physical exercise:

$$PEx_{i}=population_{i}*regular\;PEx\;participation\;\;rate_{i}$$ (4)

where population_i_ represents the total population of city i in 2019, and regular PEx participation rate_i_ represents the proportion of regular physical exercise participants of city i in 2019.

Data on the proportion of regular physical exercise participants were collected from official bulletins in the National Fitness Report, the National Fitness Development Survey Bulletin, and the National Fitness Action Program released by each province, which refers to the proportion of residents aged 7 years and above who participate in physical exercise, moderate intensity or above, for at least 30-minute 3 times or more per week. According to the General Administration of Sport of China, moderate-intensity physical exercise refers to activities that require some exertion but still allow you to speak easily during the activity, such as fast walking, dancing, leisure swimming, playing tennis, and playing golf. Moderate exercise intensity is often represented by brisk walking, and the lower limit of moderate intensity is a medium speed (4km/h).

To comprehend the general situation of the physical fitness participation of urban and rural residents in China and to evaluate the implementation effect of the national fitness strategy, the General Administration of Sport of China regularly organizes the investigation of the national fitness and issues the National Fitness Investigation Bulletin. According to the guiding opinions stemming from the investigation, each provincial General Administration of Sport collects information including the number of people who participate in physical exercise once or more a week, the number of people who regularly participate in physical exercise, the venues and facilities, and the content and the duration of physical exercise of the permanent population of cities, counties, and districts using multistage stratified random sampling.

#### 2.2.3 Control variables

With respect to control variables, social and economic factors affected the development of the epidemic, among which population density [73], economic development [55], and traffic flow [74] were important influential factors. Therefore, in this paper, we selected urban population density, per capita GDP, and the total number of public-operated buses and taxis as control variables and introduced them into the model.

The epidemic risk perception of the public helped them to take personal precautions in time and reduce unnecessary contacts, thus reducing the possibility of being infected [75, 76]. In this paper, we made use of the Baidu search index term "COVID-19" as the proxy variable of residents’ protection awareness. We converted the Baidu search index, a continuous variable, into a 0–1 dummy variable according to the mean value. If it was higher than the mean value, the value was 1, indicating high self-protection awareness of residents; otherwise, the value was 0.

In China, timely and strict public health interventions have played a crucial role in the mitigation of the COVID-19 outbreak. Referring to Huang et al. [77], we scored the public health measures implementation intensity of each city based on the epidemic prevention policy announcement issued by the health commission and converted it into a dummy variable according to the mean value. If the value was higher than the mean value, then the value was 1, indicating relatively strict prevention and control intensity; otherwise, the value was 0.

Studies have shown that population mobility does not mainly depend on the geographical distance between regions, but on the convenience of mobility, that is, the effective distance between regions [77]. Referring to Lin et al. [59], we calculated the effective distance to Wuhan, the city with the most severe epidemic in China. We retrieved the data from the Baidu migration website using big data technology.

### 2.3 Study design

First, the mean, standard deviation, maximum, and minimum values of all variables were calculated, as is shown in Table 1. Then, in order to analyze the effect of physical exercise participation on COVID-19 outcomes, we constructed a multiple linear regression, estimated by ordinary least squares (OLS) [78]. The empirical model is as follows:

$$outcomes_{i,t}=\alpha_{0}+\alpha_{1}PE_{i}+\alpha_{2}X_{i,t}+\delta_{c}+\delta_{t}+\varepsilon_{i,t}$$ (5)

where *outcomes*_*i*,*t*_ is the dependent variable, representing a series of COVID-19 outcomes of city i in date t, including morbidity, mortality, and cure rates. *PE*_*i*_ is the independent variable, which denotes the physical exercise participation of city i in 2019, i.e., the number of people who engage in physical exercise regularly. *X*_*i*_ represents a series of control variables, including urban population density, GDP per capita, effective distance to Wuhan city, dummy variable of residents’ awareness of prevention, dummy variable of public health measures, and the total number of public-operated buses and taxis. *δ*_*c*_ is a provincial fixed effect to control for provincial factors that do not vary over time, and *δ*_*t*_ is a time-fixed effect to control for temporal factors that do not vary with individuals. *ε*_*i*,*t*_ is random error term.

**Table 1:** Statistical description.

Type	Variable	Obs	Mean	Std. Dev.	Min	Max
Dependent variables	morbidity	21379	.6943053	4.698938	0	83.05714
	mortality	21379	.0058666	.0249426	0	1
	cure rate	21379	.4941528	.3372486	0	1
Independent variables	PEx	21379	3.489131	.8286374	1.558565	6.808398
Control Variables	pop density	21379	4.78546	.7807699	2.833213	7.804659
	pergdp	21379	11.10342	.6447762	8.990403	13.13379
	effective distance	21379	5.716027	1.873895	0	7.78463
	awareness	21379	.3730296	.4836212	0	1
	public health	21379	.3542682	.4830274	0	1
	public transport	21379	6.755665	1.099516	4.143135	10.48598

## 3 Result

### 3.1 Statistical description

A statistical description of the variables is shown in Table 1.

### 3.2 Influence of PEx on COVID-19 outcomes

Table 2 demonstrates the effects of physical exercise on COVID-19 outcomes, broken down as follows: columns (1)—(2) of Table 2 report the influence of PEx on COVID-19 morbidity, columns (3)—(4) report the influence of PE on COVID-19 mortality, and columns (5)—(6) report the PEx effect on COVID-19 cure. Columns (1), (3), and (5) report the results of regressions of dependent variables on independent variables alone. To avoid biased estimation results due to significant omitted variables, columns (2), (4), and (6) introduce a series of factors that also influence the variable of interest, i.e., control variables, based on columns (1), (3) and (5), the treatment of which helped to identify causal relationships. It can be seen that the coefficients of PEx in columns (1) and (2) are negative and significant at the 1% level, indicating that physical exercise participation shows a negative causal relationship with the COVID-19 incidence, i.e., participating in physical exercise significantly reduces the morbidity of COVID-19 epidemic. Similarly, PEx coefficients in columns (3) and (4) are also significantly negative, indicating that engaging in physical exercise can significantly reduce the COVID-19 mortality rate. As for the right-hand third of the Table 2, PEx coefficients in columns (5) and (6) were significantly positive, indicating that physical exercise positively affects COVID-19 cure rate.

**Table 2:** Baseline result.

	(1)	(2)	(3)	(4)	(5)	(6)
	morbidity	morbidity	mortality	mortality	cure rate	cure rate
PEx	-0.3735***	-1.1061***	-0.0370***	-0.3836***	0.0475***	0.0448***
	(0.0386)	(0.1328)	(0.0053)	(0.0177)	(0.0072)	(0.0074)
pop density		0.9413***		0.3709***	-0.0677***	-0.0713***
		(0.1616)		(0.0216)	(0.0077)	(0.0090)
pergdp		0.5577***		-0.1641***		-0.0148***
		(0.0726)		(0.0097)		(0.0040)
effective distance		-0.9325***		-0.1207***		0.0008
		(0.0228)		(0.0030)		(0.0013)
awareness		-1.0300***		-0.1498***		0.0286***
		(0.0832)		(0.0111)		(0.0046)
public health		0.5236***		-0.0035		0.0055
		(0.0883)		(0.0118)		(0.0049)
public transport		-0.9158***		-0.0104		-0.0001
		(0.0667)		(0.0089)		(0.0037)
Constant	1.9976***	5.5729***	6.2640***	8.3380***	0.6524***	0.8259***
	(0.1382)	(0.8017)	(0.0189)	(0.1071)	(0.0147)	(0.0446)
Observations	21,379	21,379	21,379	21,379	21,379	21,379
R-squared	0.053	0.136	0.174	0.279	0.480	0.482
Control Variables	NO	YES	NO	YES	NO	YES
Province FE	YES	YES	YES	YES	YES	YES
Time FE	YES	YES	YES	YES	YES	YES

Standard errors in parentheses,

*** p<0.01,

** p<0.05,

* p<0.1

### 3.3 Robustness test

First, considering that Hubei was the province with the most severe outbreak in 2020, in the robustness test, we excluded Hubei Province from the full sample to re-estimate the coefficients, and the results are reported in columns (1) to (3) of Table 3. Second, since the large-scale COVID-19 outbreak in China did not begin until late January 2020, there were no cases in most cities in January. To prevent the interference of too many zero values on the estimation results, we excluded the sample of January to re-estimate the coefficients, and the results are reported in columns (4) to (6) of Table 3. The results of the robustness test show that the regression coefficients of PEx on morbidity and mortality remained significantly negative, and those on cure rate remained significantly positive, which is consistent with the result of baseline regression, which proves the robustness of the results.

**Table 3:** Robustness.

	(1)	(2)	(3)	(4)	(5)	(6)
	Exclude Hubei	Exclude Hubei	Exclude Hubei	Exclude January	Exclude January	Exclude January
	morbidity	mortality	cure rate	morbidity	mortality	cure rate
PEx	-0.0197***	-0.4329***	0.0318***	-1.6402***	-0.3698***	0.0743***
	(0.0071)	(0.0182)	(0.0075)	(0.2128)	(0.0230)	(0.0103)
Observations	20,455	20,455	20,455	12,762	12,762	12,762
R-squared	0.360	0.295	0.479	0.205	0.281	0.534
Control Variables	YES	YES	YES	YES	YES	YES
Province FE	YES	YES	YES	YES	YES	YES
Time FE	YES	YES	YES	YES	YES	YES

Standard errors in parentheses,

*** p<0.01,

** p<0.05,

* p<0.1

### 3.4 Heterogeneity of health benefits of PEx in high-risk and low-risk areas

Further, we defined samples with cumulative case growth rates greater than the mean value as the high-risk group and those less than the mean value as the low-risk group, and performed sub-sample regressions to explore the heterogeneous PEx benefit in high- and low-risk areas. Columns (1) and (2) in Table 4 report the subsample results for the PEx effect on morbidity; columns (3) and (4) report the results for mortality; and columns (5) and (6) report that of cure rates. The results show that the coefficient of PEx is greater in high-risk areas than in low-risk areas for both morbidity, mortality, and cure rates, especially for the effect on the morbidity of the epidemic, suggesting that the health benefits of physical exercise are relatively greater and more significant when the pandemic is more severe.

**Table 4:** Heterogeneity exploration.

	(1)	(2)	(3)	(4)	(5)	(6)
	high risk	low risk	high risk	low risk	high risk	low risk
	morbidity	morbidity	mortality	morbidity	cure rate	cure rate
PEx	-1.8776***	-0.0037***	-0.3982***	-0.3492***	0.0807***	0.0193**
	(0.2770)	(0.0006)	(0.0269)	(0.0234)	(0.0117)	(0.0091)
Observations	10,686	10,693	10,686	10,693	10,686	10,693
R-squared	0.204	0.564	0.257	0.322	0.578	0.308
Control Variables	YES	YES	YES	YES	YES	YES
Province FE	YES	YES	YES	YES	YES	YES
Time FE	YES	YES	YES	YES	YES	YES

Standard errors in parentheses,

*** p<0.01,

** p<0.05,

* p<0.1

## 4 Discussion

There are many factors that influence COVID-19 outcomes, including public health interventions, vaccination availability and rates, demographic characteristics, environmental variables, health care resources, etc. [52–54, 58–61, 63]. In our research, we constructed a two-way fixed effect model of multiple linear regression to empirically investigate the effects of physical exercise on a series of COVID-19 outcomes using prefecture-daily data related to PEx and COVID-19 from January 1 to March 17, 2020 in mainland China. The results show that there is relatively lower COVID-19 morbidity, mortality, and a higher cure rate during the pandemic in areas with a higher proportion of engaging in regular physical exercise before it, implying that PEx does have a significant effect on COVID-19 outcomes.

Our study expands the body of research on the health benefits of physical exercise, which has been demonstrated in existing studies to be effective not only in reducing the risk of chronic diseases [79, 80] but also in improving autoimmunity [81] and also has a positive effect on the suppression of emerging infectious diseases [46]. Furthermore, our study is consistent with the results of the existing literature exploring physical exercise effect on pandemics, demonstrating the positive role of PEx in the pandemic [82–84], with the difference being that we focus on the effects of physical exercise before the outbreak on the health outcomes during it, i.e., we pay more attention to the long-term effects of physical exercise. Moreover, in contrast to the existing literature, in addition to infection and mortality rates, we also discuss the influence of PEx on COVID-19 cure rates.

Further, we also explore the heterogeneity of the physical exercise effect in areas with different risk levels, and the results show that the influence of PEx is more significant in high-risk areas, in terms of morbidity, mortality, and cure rates. In particular, the difference in PEx benefits on morbidity is greatest between high- and low-risk areas, suggesting that the effect of PEx on morbidity is most pronounced in high-risk areas. This result suggests that participating in physical exercise regularly before such an outbreak makes significant health effects, especially in the case of a more severe epidemic; therefore, we should actively engage in physical exercise to reduce risks to our health when a pandemic comes.

However, there are still some limitations that need to be explored in the future. First, restricted by the available data and the period of the selected sample in this study, we do not consider the effect of vaccination. In the following studies, we will attempt to obtain data sets for longer periods, as well as vaccination-related data. In addition, the current results fail to control individual factors that would influence COVID-19 outcomes, so we attempt to match region-level data with micro-individual data to control for the potential impact of individual information on epidemic outcomes and to explore more heterogeneous results for individual-level characteristics such as gender and age. Third, limited by the availability of physical exercise-related data, we are unable to capture information on exercise intensity, which is also a very important influential factor. In the following studies, we will try to apply data mining techniques to access exercise big data to better identify information on exercise duration, intensity, and frequency to obtain more accurate conclusions.

## 5 Conclusion

In this article, we constructed a two-way fixed effects model of multiple linear regression, collected daily prefectural data related to physical exercise and the COVID-19 pandemic in mainland China from January 1 to March 17, 2020, and estimated the health benefit of engaging in regular physical exercise before the pandemic on a series of COVID-19 outcomes during the pandemic. We found that there is relatively lower COVID-19 morbidity, mortality, and a higher cure rate in areas with a higher proportion of individuals engaging in regular physical exercise before the pandemic, implying that PEx has a significant positive effect on COVID-19 outcomes. Finally, we also examined the heterogeneity of the health benefit of physical exercise in areas with different risk levels, and the results showed that the effect of PEx was more pronounced in high-risk areas in terms of morbidity, mortality, and cure rates.

## Supporting information

S1 Data (XLSX)
